# Ethnic differences in BMI among Dutch adolescents: what is the role of screen-viewing, active commuting to school, and consumption of soft drinks and high-caloric snacks?

**DOI:** 10.1186/1479-5868-6-23

**Published:** 2009-04-21

**Authors:** Amika S Singh, Mai JM Chinapaw, Johannes Brug, Stef PJ Kremers, Tommy LS Visscher, Willem van Mechelen

**Affiliations:** 1 VU University Medical Center, EMGO-Institute, Department of Public and Occupational Health, Amsterdam, the Netherlands; 2 VU University Medical Center, EMGO-Institute, Amsterdam, the Netherlands; 3 Department of Health Education and Health Promotion, Universiteit Maastricht, Maastricht, the Netherlands; 4 Vrije Universiteit, Institute for Health Sciences, Amsterdam, the Netherlands

## Abstract

**Background:**

The threats posed by the rising prevalence of overweight and obesity on public health have been widely acknowledged. Several population groups, which deserve special attention because of their higher prevalence rates, have been identified. These include adolescents and ethnic sub-groups. The aim of the present study was twofold: (1) to assess ethnic differences in body mass index (BMI) and in behaviours that are related to both energy intake and energy expenditure, and (2) to examine whether these behaviours explain the relationship between ethnicity and BMI.

**Methods:**

We conducted a cross-sectional data analysis among 957 Dutch adolescents (mean age = 12.7 years). Body height and weight were measured using a standardized protocol. Adolescents completed a questionnaire on screen-viewing behaviour, physical activity, consumption of sugar-containing beverages, and consumption of high-caloric snacks.

**Results:**

In our study sample 121 adolescents (= 13%) were of Non-Western origin. BMI was significantly higher in Non-Western adolescents (boys: 19.9 kg/m^2^, SD = 3.0, girls: 20.9 kg/m^2^, SD = 3.8) compared to Dutch adolescents (boys: 18.4 kg/m^2^, SD = 2.8, girls: 19.0 kg/m^2^, SD = 3.0). Our results show that time spent on television viewing, active commuting to school, and consumption of fruit juices partially mediated the association between BMI and ethnicity.

**Conclusion:**

Behaviours related to both energy expenditure and energy intake may contribute to the ethnic differences in BMI in adolescents and should be considered when tailoring overweight prevention programs to ethnic subpopulations.

**Trial registration:**

International Standard Randomised Controlled Trial Number ISRCTN87127361

## Background

The threats that the rising prevalence of overweight and obesity pose on public health have been widely acknowledged [1]. Several high-risk groups have been identified with increased prevalence rates of overweight and obesity, among which adolescents [2] and ethnic subgroups [3,4].

Onset of overweight during adolescence is particularly worrisome. Not only is it a strong predictor for increased risk of overweight in adulthood [5], but overweight status during adolescence is also associated with increased morbidity and mortality in later life [6]. Moreover, during adolescence health behaviours, among which energy balance-related behaviours (e.g. sedentary behaviour, physical activity, consumption of soft drinks and energy-dense snacks), are subject to important changes [7]. Unfavourable behavioural patterns established during this age period are vital in the development of adult health behaviours [8], and are therefore important for weight status in adulthood.

The burden of the obesity epidemic is not equally shared by all segments of the population. Several studies show that the prevalence of obesity is disproportionally high among ethnic subgroups and among people with lower socio-economic position, both in the adult population [9] and in youth [4,10-14]. Although socio-economic class and ethnicity are strongly correlated, it is established that the latter is independently associated with health [15]. The factors that govern this independent association may be related to cultural differences in underlying causes of excessive weight gain, overweight and obesity.

Although weight gain is influenced by many factors, it is clear that energy balance-related behaviours are playing a major role in the onset of overweight. Risk behaviours that are associated with excessive weight gain in adolescents are sedentary behaviour (especially screen-viewing behaviour), lack of physical activity, and consumption of high-caloric foods and beverages [16]. Population data suggest that ethnic disparities with regard to overweight are established during adolescence [13,17]. It thus seems likely that these disparities reflect behavioural differences between ethnic groups that arise in the transition from childhood to adolescence [7]. Understanding how the different energy balance-related behaviours contribute to ethnic disparities in BMI in adolescents may enable us to target preventive intervention programs to specific subgroups. In the present cross-sectional study, we assessed ethnic differences in body mass index (BMI) and energy balance-related behaviours among a sample of adolescents attending prevocational secondary education in the Netherlands. Furthermore, we examined whether and which behaviours mediated the association between ethnicity and BMI.

## Methods

### Design and subjects

We conducted a cross-sectional data analysis, using baseline data collected in September 2003 from adolescents participating in a randomized controlled study, i.e. the Dutch Obesity Intervention in Teenagers (DOiT). Details on the aim, design and methods of DOiT have been published elsewhere [18]. All first year students of the 18 participating schools were invited for participation in DOiT; no inclusion criteria were set for students to participate. Written informed consent was obtained from all students and their parents. The Medical Ethical Committee of the VU University Medical Center approved the study protocol.

### Measures

#### Body composition

Trained research assistants performed anthropometric measurements, according to a standardized protocol. Body height was measured with an accuracy of 1 mm with a portable stadiometer (Seca 225). Body weight was measured and recorded within 0.1 kg with a calibrated electronic flat scale (Seca 888). Students were dressed in underwear during all measurements. We calculated body mass index (BMI) and used age and sex specific cut-off points to classify weight status according to the criteria of the International Obesity Task Force [19].

#### Questionnaire

##### General

Demographic and behavioural data were derived from a questionnaire that adolescents completed in the classroom. A teacher and/or member of the DOiT-team supervised the completion of the questionnaires.

##### Ethnicity

Data on ethnicity were collected by self-report according to a standard question on the country of birth of the parents (*"Where was your mother/father born?"*). According to Statistics Netherlands [20], an adolescent is considered to be of Dutch ethnicity if both parents were born in the Netherlands. An adolescent with at least one parent born in a foreign country is considered to be of foreign ethnicity. This definition, however, does not always cover the cultural differences that may importantly influence behaviour. Therefore we used an alternative definition [20], to differentiate between Dutch adolescents and adolescents from Non-Western origin. Pursuant to this definition, adolescents with at least one parent born in Turkey, Africa, Latin America or Asia were classified as Non-Western immigrants. Adolescents with at least one parent born outside the Netherlands, but inside Europe (including former Yugoslavia and Soviet Union), North America, Oceania, Indonesia or Japan were classified as 'Western immigrants' (n = 40). In the following we use the term 'ethnicity' with subdivisions 'Dutch' and 'Non-Western' to refer to the ethnic differences as defined above.

##### Behaviour

The following energy balance-related behaviours were addressed: (1) consumption of sugar-containing beverages (i.e. consumption of soft drinks and fruit juices [millilitres/day]), (2) consumption of high-caloric snacks (i.e. consumption of savoury snacks and sweet snacks [portions/day]), (3) screen-viewing behaviour (i.e. time spent on television viewing and computer use [minutes/day]), and (4) physical activity (i.e. active transport to school, participation in organized sports, and participation in unorganized sports [minutes/day]). Since there are no validated Dutch questionnaires addressing the behaviours selected for our study, we adapted other validated questionnaires on dietary intake [21,22], screen-viewing behaviour [23], and physical activity [24] in adolescents. The structure of the questionnaire was equal for all behaviours. For a more detailed description of the questionnaire see appendix 1. The questionnaire was pre-tested for clarity and duration, among adolescents not participating in the study by means of cognitive interviewing [25].

Frequency and quantity were multiplied in order calculate daily consumption (separately for weekdays and weekend days). Weekday consumption (multiplied by 5) and weekend day consumption (multiplied by 2) were summed up and divided by 7 to calculate mean daily consumption. Since some subjects reported unrealistic values with regard to values above the 95^th ^percentile were recoded as the value of the 95^th ^percentile.

### Statistical analyses

We calculated descriptive statistics for anthropometric measures, and assessed differences between Dutch and Non-Western adolescents in these variables (Kolmogorov; Smirnov Z), and in the prevalence of overweight/obesity (Pearson; Chi-Square). As most of the behavioural variables were skewed, we calculated means and medians, and the 25^th ^and 75^th ^percentiles for all behaviours. We examined differences between Dutch and Non-Western adolescents in energy balance-related behaviours with non-parametric Mann-Whitney Tests.

**Figure 1:**
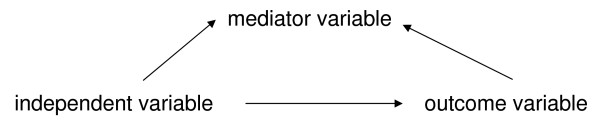
**Mediated relationship (according to Baron and Kenny **[26])

**Figure 2:**
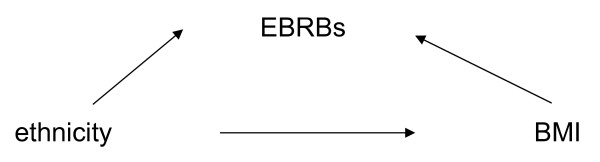
Energy balance-related behaviours (EBRBs) as a mediator variable of the relationship between BMI and ethnicity (Dutch versus Non-Western)

To test for mediation by sedentary behaviour, physical activity, consumption of soft drinks and energy-dense snacks we followed the principles outlined by Baron and Kenny [26]. Consistent with Baron and Kenny's conditions (see figures 1 and 2), a variable functions as a mediator when it meets the following four criteria: (1) the independent variable (ethnicity) must be associated with the outcome variable (BMI) (crude model); (2) the independent variable (ethnicity) must be associated with the mediator variables (energy balance-related behaviours); (3) the mediator variables (energy balance-related behaviours) must be associated with the outcome variable (BMI) after controlling for the independent variable (ethnicity) (adjusted models); and (4) the relationship between the independent variable (ethnicity) and the outcome variable (BMI) is significantly diminished when adjusting for the mediator variables (energy balance-related behaviours).

We calculated three (multiple) regression equations [27], with ethnicity as an independent dichotomous variable (Dutch = 0, Non-Western = 1). Furthermore, a Sobel Test [28] was conducted to assess if the relationship between ethnicity and BMI significantly decreased after energy balance-related behaviours were added to the regression model (criterion 4).

The signs and magnitudes of the regression coefficients indicate whether or not the third variable is a mediator or if it must be interpreted as a suppressor. If both regression coefficients share the same sign, a decreased adjusted association indicates (partial) mediation, while an increased adjusted association or an opposite sign indicates suppression/inconsistent mediation [29].

We report the unstandardized Beta statistics from linear regression that can be interpreted as the difference between Dutch and Non-Western adolescents.

Missing data (due to absence at the measurement day due to illness, doctor's visit, et cetera) on demographics, body composition, and energy balance-related behaviours were not imputed. In case data on weekend consumption (or activity) were not complete, weekday data were used to calculate the mean score for the daily score, and vice versa.

Significance levels were set at *p *≤ .05. All analyses were conducted using SPSS version 13.0.

## Results

### Participation and completion rate

A total of 18 schools, located throughout the Netherlands, participated in the study. Of 1323 invited adolescents, 1108 (84%) returned both the parental and student informed consent. Complete data from anthropometric measurements and questionnaires were obtained from 1014 (92%) adolescents. After exclusion of the group of Western immigrants, data of 957 (94%) adolescents were available for the present analyses.

### Ethnic differences in body composition

In table 1 anthropometric data of our study sample are presented, stratified for gender and ethnicity. Mean age of the sample was 12.7 (SD = .5) years. Approximately 13% (N = 121) of all adolescents were of Non-Western origin. BMI and prevalence rates of obesity differed significantly between Dutch and Non-Western boys and girls, with significantly lower BMI and overweight/obesity prevalence rates in Dutch adolescents.

**Table 1:** Demographic and anthropometric variables in Dutch and Non-Western adolescents

	**boys**			**girls**		
Characteristics	Dutch (n = 410)	Non-Western (n = 64)	*P* Value^a^	Dutch (n = 426)	Non-Western (n = 57)	*P* Value*

age, y	12.7 (.4)	13.0 (.7)	.039	12.6 (.5)	12.8 (.5)	.054
height, cm	159.3 (8.2)	157.9 (8.1)	.163	158.3 (7.3)	156.1 (7.7)	.182
weight, kg	47.0 (9.9)	49.9 (9.6)	.169	47.8 (9.5)	51.2 (12.0)	.066
BMI, kg/m^2^	18.4 (2.8)	19.9 (3.0)	.000	19.0 (3.0)	20.9 (3.9)	.003
overweight, No. (%)† obese, No. (%)^b^	56 (13.7) 5 (1.2)	13 (20.3) 4 (6.3)	.007	64 (15.0) 11 (2.6)	14 (24.6) 6 (10.5)	.001

Data are presented as mean (SD) unless stated otherwise

* comparing Dutch and Non-Westerners, using the Kolmogorov Smirnov Test (all variables, except for %overweight/%obese), and the Pearson Chi-square (%overweight/% obese)

† using cut-off values described by Cole et al. [19]

### Energy balance-related behaviours

In table 2 and 3 means and medians for all energy balance-related behaviours are shown. In the text below we present medians.

**Table 2:** Energy balance-related behaviours (EBRBs) in Dutch and Non-Western boys

		**Dutch**		**Non-Western**		
EBRBs	n	mean (std)	median (25^th ^– 75^th ^percentile)	mean (std)	median (25^th ^– 75^th ^percentile)	*P *Value*

**Screen-viewing behaviour, min/day**						

television viewing	451	164 (96.8)	141 (94.3 – 212)	191 (108)	180 (86.8 – 267)	.074

computer use	433	123 (86.0)	98 (60.0 – 178)	141 (80.7)	129 (77.1 – 30.0)	.275

**Physical activity, min/day**						

active transport to school	469	38.0 (29.6)	30.0 (14.0 – 60.0)	28.42 (24.9)	30.0 (10.0 – 30.0)	.006

organized sports	322	33.8 (19.6)	32.1 (18.6 – 42.9)	34.2 (20.3)	28.6 (21.4 – 49.3)	.275

unorganized sports	245	93.3 (111)	51.4 (25.7 – 111)	126 (1434)	164 (32.1 – 159)	.912

**Consumption of sugar-containing drinks, ml/day**						

soft drinks	375	890 (710)	685 (347 – 1251)	1041 (780)	918 (153 – 1438)	.331

fruit juices	392	330 (391)	171 (28.6 – 468)	507 (499)	386 (10.2 – 788)	.025

**High-caloric snack consumption, portions/day**						

savoury snacks	415	.59 (.51)	.43 (.29 – .86)	.66 (.54)	.43 (0.29 – 1.00)	.660

sweet snacks	427	1.58 (1.17)	1.00 (.71 – 2.00)	1.26 (.97)	.93 (.54 – 2.00)	.141

*comparing Dutch and Non-Western adolescents, using the Mann-Whitney Test

**Table 3:** Energy balance-related behaviours (EBRBs) in Dutch and Non-Western girls

		**Dutch**		**Non-Western**		
EBRBs	n	mean (std)	median (25^th ^– 75^th ^percentile)	mean (std)	median (25^th ^– 75^th ^percentile)	*P *Value*

**Screen-viewing behaviour, min/day**						

television viewing	461	142 (86.4)	120 (77.1 – 180)	1867 (112)	161 (93.8 – 272)	.033

computer use	429	89 (67.6)	68.6 (34.29 – 120)	102 (71.2)	81.4 (60.0 – 137)	.529

**Physical activity, min/day**						

active transport to school	480	36.8 (28.3)	30.0 (18.0 – 60.0)	27.3 (24.9)	20.0 (10.0 – 30.0)	480

organized sports	305	23.5 (16.4)	19.3 (10.7 – 30.0)	26.1 (18.6)	18.2 (11.8 – 37.5)	305

unorganized sports	204	53.4 (75.6)	34.3 (17.1 – 51.4)	75.4 (93.6)	34.3 (17.1 – 90.0)	204

**Consumption of sugar-containing drinks, ml/day**						

soft drinks	395	774 (634)	657 (281 – 1142)	918 (700)	714 (439 – 1189)	395

fruit juices	396	326 (342)	200 (57.1 – 486)	476 (462)	323 (108 – 641)	396

**High-caloric snack consumption, portions/day**						

savoury snacks	453	.58 (.51)	.43 (.14 – 86)	.77 (.59)	.57 (.29 – 1.00)	.044

sweet snacks	464	1.38 (.98)	1.00 (.57 – 2.00)	1.23 (1.10)	.86 (.43 – 2.00)	.094

*comparing Dutch and Non-Western adolescents, using the Mann-Whitney Test

#### Screen-viewing behaviour

Non-Western adolescents reported on average to watch more television than their Dutch peers. The difference of approximately 39 minutes per day between Dutch boys (141 minutes) and their Non-Western peers (180 minutes) was not statistically significant (p = .074). Non-Western girls reported on average 161 minutes per day of television viewing versus 120 minutes per day in Dutch girls (p = .033). Computer use was also higher in Non-Western adolescents. Differences in computer use between Dutch and Non-Western adolescents were not statistically significant.

#### Physical activity

Dutch adolescents reported on average more active commuting to school than their Non-Western peers. Differences were statistically significant both in boys (p = .037) and girls (p = .006). Differences between Dutch and Non-Western adolescents with regard to sports participation, both organized and unorganized, were not statistically significant.

#### Sugar-containing beverage consumption

Soft drink and fruit juice consumption was lower in Dutch than in Non-Western adolescents. Differences were not statistically significant.

#### High-caloric snack consumption

Both Dutch and Non-Western boys reported to consume on average 0.43 portions of savoury snacks and approximately 1.0 portions of sweet snacks per day. Non-Western girls reported to consume significantly more savoury snacks (.57 portions per day) than Dutch girls (.43 portions per day) (p = .005). Consumption of sweet snacks was higher among Dutch girls (1.00 portions per day versus .86 portions per day; p = .094).

### Exclusion of Western immigrants from the analyses

Comparing energy balance-related behaviours of Dutch adolescents with Non-Western and Western immigrants showed no consistent pattern, i.e. Western immigrants resemble Dutch adolescents in some behaviours, in other behaviours they resemble Non-Western adolescents, and in some behaviours none of these two groups (table 4).

**Table 4:** Differences between Dutch, Non-Western immigrants, and Western immigrants with regard to energy balance-related behaviours. Data are presented as mean (SD)

	Dutch	Non-Western immigrants	Western immigrants
**boys**			
television viewing	164 (96.8)	191 (108)	222 (148)
computer use	123 (86.0)	141 (80.7)	115 (39.2)
soft drink consumption	890 (710)	1041 (780)	930 (972)
fruit juice consumption	330 (391.29)	507 (499)	451 (540)
active transport to school	38 (29.6)	28.4 (24.9)	26 (11.4)
savoury snack consumption	.59 (.51)	.66 (.54)	.69 (.68)
sweet snack consumption	1.58 (1.17)	1.26 (.97)	1.60 (1.46)
			
**girls**			
television viewing	142 (86.4)	187 (112)	139 (52.9)
computer use	89.0 (67.6)	102 (71.2)	104 (104)
soft drink consumption	774 (634)	918 (700)	706 (800)
fruit juice consumption	326 (342)	476 (462)	290 (410)
active transport to school	36.8 (28.30)	27.3 (24.9)	35.3 (47.4)
savoury snack consumption	.58 (.51)	.77 (.59)	.71 (.25)
sweet snack consumption	1.38 (.98)	1.23 (1.10)	1.71 (.49)

### Mediation analysis: relationship between ethnicity and BMI mediated by energy balance-related behaviours (EBRBs)

Ethnicity was significantly associated with BMI, indicating that Non-Western boys and girls were more likely to have a higher BMI than Dutch adolescents (table 5, criterion 1). Non-Western boys and girls reported to watch more television, consume more fruit juices and savoury snacks (girls only), and spend less minutes on active commuting to school when compared to Dutch adolescents (table 5, criterion 2).

**Table 5:** Results of testing the mediational model: energy balance-related behaviours (EBRBs) as mediators of the relationship between ethnicity and BMI (criteria 1 and 2)

	**boys**		**girls**	
	unstandardized regression coefficient (95% CI)	SE*	unstandardized regression coefficient (95% CI)	SE*

**BMI: ethnicity (criterion 1)†**				

BMI	1.54 (.80 – 2.28)	.37	1.86 (1.00 – 2.72)	.44

				

**EBRBs: ethnicity (criterion 2)‡**				

**screen-viewing behaviour**				

television viewing	26.75 (.30 – 53.21)	13.46	44.28 (18.73 – 69.82)	13.00

computer use	17.63 (-6.58 – 41.84)	12.32	13.14 (-6.88 – 33.16)	10.18

**physical activity**				

active transport to school	-9.60 (-17.27 – -1.93)	3.90	-9.46 (-17.26 – -1.66)	3.97

organized sports	.41 (-6.52 – 7.33)	3.52	2.62 (-6.26 – 11.49)	4.51

unorganised sports	32.52 (-6.69 – 71.73)	19.91	22.07 (-8.97 – 53.12)	15.75

**sugar-containing beverage consumption**				

soft drinks	151.03 (-73.48 – 375.54)	114.12	143.79 (-60.01 – 347.59)	103.66

fruit juices	177.13 (54.22 – 300.05)	62.52	150.27 (38.07 – 262.47)	57.07

**high-caloric snack consumption**				

savoury snacks	.07 (-.08 – .22)	.08	.19 (.03 – .34)	.08

sweet snacks	-.32 (-.65 – .01)	.17	-.15 (-.44 – .14)	.17

*SE = standard error

†regression model with BMI as criterion variable and ethnicity as predictor variable

‡regression model with EBRBs as criterion variable and ethnicity as predictor variable

**Figure 3:**
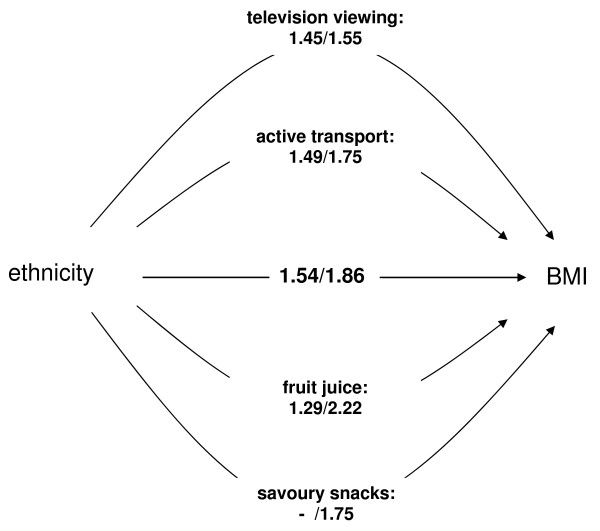
**Model depicting the associations between ethnicity (independent variable) and BMI (outcome variable), mediated by energy balance-related behaviours (mediator variables)**. Correlation coefficients are presented in parentheses for boys and girls, respectively

Results of multiple regression models adjusted for EBRBs as well as the Sobel Test (table 6, criteria 3 and 4) showed that in boys the association between BMI and ethnicity was significantly mediated by television viewing, active transport to school, and consumption of fruit juices (figure 3). In girls, the association between BMI and ethnicity was significantly mediated by television viewing, active transport to school, consumption of fruit juices, and consumption of savoury snacks (figure 3). Adding all EBRBs that met all four criteria resulted in a significant decrease of the regression coefficient, both in boys and girls (figure 3).

**Table 6:** Results of testing the mediational model: energy balance-related behaviours (EBRBs) as mediators of the relationship between ethnicity and BMI (criteria 3 and 4)

	unstandardized regression coefficient (95% CI)	% mediation	SE*	Sobel z-value	P value (Sobel)
**boys**					

**BMI: ethnicity and EBRBs (criteria 3 and 4)^†^**					

television viewing	1.45 (.69 – 2.21)	5.8	.39	2.73	.006

active transport to school	1.49 (.75 – 2.23)	3.3	.38	2.81	.005

fruit juices consumption	1.29 (.43 – 2.16)	16.2	.44	2.35	.02

savoury snacks consumption	NA	NA	NA	NA	NA

**BMI: ethnicity and all EBRBs**‡	1. 16 (.28 – 2.05)	24.7	.45	2.15	.03

					

**girls**					

**BMI: ethnicity and EBRBs (criteria 3 and 4)^†^**					

Television viewing	1.55 (.68 – 2.42)	16.7	.44	2.66	.008

active transport to school	1.75 (.87 – 2.62)	5.9	.44	2.86	.004

fruit juices consumption	2.22 (1.23 – 3.21)	S	.50	3.02	.003

*savoury snacks consumption*	1.75 (.89 – 2.61)	5.9	.44	2.86	.004

**BMI: ethnicity and all EBRBs**‡	1.39 (.42 – 2.37)	25.3	.50	2.28	.02

*SE = standard error

†regression model with BMI as the criterion variable and EBRBs and ethnicity as predictor variables

‡regression model with BMI as the criterion variable and television viewing, active transport to school, fruit juices, and savoury snacks (girls only)

NA = not applicable

S = suppression effect

## Discussion

To our best knowledge, this is the first study exploring whether ethnic differences in objectively measured BMI can be explained by energy balance-related behaviours, taking into account both sides of the energy balance, using mediation analysis.

Our results show that Non-Western adolescents were more likely to have a higher BMI than Dutch adolescents, confirming results of other studies among European children and adolescents [4,14,30-32]. Furthermore, we examined whether ethnic differences in energy balance-related behaviours partially explained the relationship between ethnicity and BMI and found that behaviours related to energy expenditure (television viewing and active transport behaviour) and energy intake (consumption of fruit juices and consumption of savoury snacks in girls) partly explained the ethnic disparities in BMI.

Our results suggest that differences in BMI between Dutch and Non-Western adolescents can partly be attributed to the larger amount of time Non-Western adolescents spend on television viewing. Our findings are consistent with previous cross-sectional studies, suggesting that time spent on television viewing is associated with a less favourable body composition [33,34] and higher prevalence rates of obesity [35] in youth. Several longitudinal data provide evidence that time spent on television viewing predicts overweight and/or obesity in adulthood and television viewing during childhood and adolescence precedes overweight and/or obesity in adulthood [36,37]. Andersen et al. [34] reported that Non-Hispanic black boys and girls had the highest rates of excessive television viewing (≥ 4 hours per day), but did not assess mediation effects of television viewing on the association between ethnicity and body composition. Previous evidence, derived from a sample of German children (aged 5–6 years), also indicates that television viewing is as an important determinant of ethnic differences in BMI [31].

Furthermore, our study findings indicate that differences with regard to commuting actively to school between Dutch and Non-Western adolescents partly explain the ethnic differences with regard to BMI in our study sample. Although the average difference per day was small (i.e. 9–10 minutes), such a small difference can have a significant impact on body composition over longer periods of time [38].

Declines in active commuting to school (i.e. the decrease in number of children actively commuting to school and the average distance travelled by bicycle and walking) and increases in overweight have both occurred at the same time [16,39]. In line with our findings, results of a cross-sectional study among Dutch adolescents show that students who used their bicycle for transportation were more likely to be native Dutch [40]. However, there is still lack of experimental evidence to prove the causal link between declines of physical activity levels and the increasing prevalence of overweight, and a recent longitudinal study report suggests that excessive time spent in sedentary behaviour may be more important than (medium and high intensity) physical activity [37].

Fruit juice consumption partly explained the ethnic differences in BMI in boys. This is in line with two recent studies that found sweetened fruit juices to be dose-response associated with a higher risk of weight gain in adults [41] and overweight and obesity in schoolchildren [42]. In girls fruit juice consumption led to a significant increase of the regression coefficient (suppression). Since Non-Western girls reported to consume significantly more fruit juice than Dutch girls, we would have expected that adjusting the crude model for consumption of fruit juices would lead to a decrease of the regression coefficient. This finding may reflect ethnic differences with regard to the rationale behind fruit juice consumption. In Non-Western girls, unlike Dutch girls, consumption of fruit juices may indicate an overall healthier diet [43].

Our findings suggest that differences in BMI between Dutch and Non-Western girls can partly be attributed to the higher consumption of savoury snacks of Non-Western girls. Reviewing the literature [44] there is no clear association between energy intake or food composition and body composition in adolescents. However, in combination with high levels of TV viewing, a relationship between snacking and body composition was found [45]. Additional analyses revealed also a positive significant association between television viewing and consumption of savoury snacks among girls of our study sample (Pearson's *r *= .23, p = .000).

We were not able to fully explain ethnic differences in BMI by all energy balance-related behaviours. The unexplained remaining difference may be contributed to the interaction of biological and social factors with an environment that includes few opportunities for being physically active and an overabundance of other high-caloric foods or beverages [46].

### Strength and limitations

A major strength of the present study is that we were able to analyze data of a relatively large study sample, with an equal gender distribution and a representative sub sample of Non-Western adolescents. Since misreporting of weight is very common, especially among overweight and obese adolescents [47-49], the quality and consistency of our anthropometric data collection is also a strong element of our study. Another strength is that we have considered a large set of behaviours that have widely been associated with overweight and obesity in children and adolescents.

Some limitations of this study are worth noting when interpreting our results. Since we analyzed cross-sectional data, cause-effect relationships between energy balance-related behaviours and BMI cannot be assumed. Randomized controlled trials are needed to gain more insight in the nature of these relationships. Besides, the assessment of behavioural variables was based on self-report, a common drawback that we share with many other studies in the field. We adapted validated questionnaires to our study population and specific energy balance-related behaviours, but did not support assessment by any objective measurement. Objective behavioural measures however often are not feasible in large epidemiological studies. In consequence, we are not able to quantify the amount of over-reporting or under-reporting. When interpreting our results one must bear in mind that especially overweight adolescents are likely to underreport energy intake and overreport physical activity [16,50].

Our study provides useful insights into the prevalence rates of overweight and obesity in Dutch and Non-Western adolescents and underlying behavioural determinants that may partly be responsible for ethnic disparities in BMI. Our findings indicate that behaviours related to both energy expenditure and energy intake may contribute to the ethnic differences in body composition that we found in our study population. Taking into account differences in energy balance-related behaviours in ethnic subpopulations help to improve targeted or tailored interventions for preventing overweight in adolescents. Therefore, interventions aimed at Non-Western subgroups should specifically focus on promoting active transport and discouraging television viewing.

## Competing interests

The authors declare that they have no competing interests.

## Authors' contributions

AS, MC, JB, SK, TV, and WvM provided support in the design of the study and contributed intellectual input into the main ideas of this paper. AS designed and coordinated the implementation of the intervention. She supervised data-collection, analysed data, and drafted the manuscript. All authors contributed to the further writing of the manuscript. MC, JB, and WvM obtained financial support. AS will act as guarantor of the paper.

## Appendix 1

Questionnaire used to examine energy balance-related behaviour.

### 1. Questions on screen-viewing behaviour

How many days a week do you watch television/use the computer? Think about last week when answering the question. [Answering categories: none, 1, 2, 3, 4, 5, 6, every day]

How long on average did you watch television/use the computer on a usual (school/weekend)day? Think about a usual school day in the last week when answering the question. [Answering categories: open answer (hours and minutes/day)]

### 2. Questions on physical activity – active transport to school

How many days did you go to school by bicycle/on foot last week? [Answering categories: none, 1 day, 2 days, 3 days, 4 days, 5 days]

How many minutes does it usually take for you to bike/walk to school? Think about a usual school day in the last week when answering the question. [Answering categories: open answer (hours and minutes)]

### 3. Questions on consumption of sugar-containing beverages

How many days did you drink soft drinks/fruit juices last week? [Answering categories: none, 1, 2, 3, 4, 5, 6, every day]

On days that you drank soft drinks/fruit juices, how many glasses/bottles/cans did you drink (weekday/weekend day)? [Answering categories: open answers (glasses/day, bottles/day, cans/day)]

### 4. Questions on consumption of high-caloric snacks

How many days did you eat savoury snacks/sweets last week? [Answering categories: none, 1, 2, 3, 4, 5, 6, every day]

When you eat savoury snacks/sweets, how many portions do you usually eat? Think about last week when answering the question. [Answering categories: open answers (portions/day)]
