# Correction: Ethnic differences in BMI among Dutch adolescents: what is the role of screen viewing, active commuting to school, and consumption of soft drinks and high-caloric snacks?

**DOI:** 10.1186/1479-5868-6-40

**Published:** 2009-07-07

**Authors:** Amika S Singh, Mai JM Chinapaw, Johannes Brug, Stef PJ Kremers, Tommy LS Visscher, Willem van Mechelen

**Affiliations:** 1 VU University Medical Center, EMGO-Institute, Department of Public and Occupational Health, Amsterdam, the Netherlands; 2 VU University Medical Center, EMGO-Institute, Amsterdam, the Netherlands; 3 Department of Health Education and Health Promotion, Universiteit Maastricht, Maastricht, the Netherlands; 4 Vrije Universiteit, Institute for Health Sciences, Amsterdam, the Netherlands

## Correction

Since publication of our article [1] we have noticed an error. The arrows in figure 1 and 2 [2] from mediator variable to outcome variable and EBRBs and BMI respectively, are pointing in the wrong direction and the correct versions of the figures are now provided with this article.

**Figure 1:**
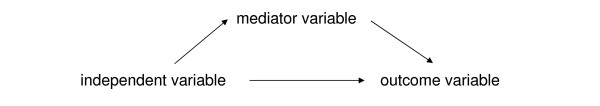
**Mediated relationship (according to Baron and Kenny **[2]**)**.

**Figure 2:**
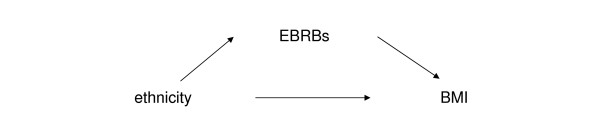
**Energy balance-related behaviours (EBRBs) as a mediator variable of the relationship between BMI and ethnicity (Dutch versus Non-Western)**.
